# Comparison of Postpartum Opioid Prescriptions Before vs During the COVID-19 Pandemic

**DOI:** 10.1001/jamanetworkopen.2023.6438

**Published:** 2023-04-03

**Authors:** Shelby R. Steuart, Emily C. Lawler, Grace Bagwell Adams, Hailemichael Shone, Amanda J. Abraham

**Affiliations:** 1 Department of Public Administration and Policy, School of Public and International Affairs, University of Georgia, Athens; 2 Department of Health Policy and Management, College of Public Health, University of Georgia, Athens; 3 Irsay Institute, Indiana University, Bloomington

## Abstract

**Importance:**

The COVID-19 pandemic substantially disrupted routine health care and exacerbated existing barriers to health care access. Although postpartum women frequently experience pain that interferes with activities of daily living, which is often successfully treated with prescription opioid analgesics, they are also at high risk for opioid misuse.

**Objective:**

To compare postpartum opioid prescription fills after the onset of the COVID-19 pandemic in March 2020 with fills before the pandemic.

**Design, Setting, and Participants:**

In this cross-sectional study of 460 371 privately insured postpartum women who delivered a singleton live newborn between July 1, 2018, and December 31, 2020, postpartum opioid fills before March 1, 2020, were compared with fills after March 1, 2020. Statistical analysis was performed from December 1, 2021, to September 15, 2022.

**Exposure:**

COVID-19 pandemic onset in March 2020.

**Main Outcomes and Measures:**

The main outcome was postpartum opioid fills, defined as patient fills of opioid prescriptions during the 6 months after birth. Opioid prescriptions were explored in terms of 5 measures: mean number of fills per person, mean filled morphine milligram equivalents (MMEs) per day, mean days supplied, percentage of patients filling a prescription for a schedule II opioid, and percentage of patients filling a prescription for a schedule III or higher opioid.

**Results:**

Among 460 371 postpartum women (mean [SD] age at delivery, 29.0 [10.8] years), those who gave birth to a single, live newborn after March 2020 were 2.8 percentage points more likely to fill an opioid prescription than expected based on the preexisting trend (forecasted, 35.0% [95% CI, 34.0%-35.9%]; actual, 37.8% [95% CI, 36.8%-38.7%]). The COVID-19 period was also associated with an increase in MMEs per day (forecasted mean [SD], 34.1 [2.0] [95% CI, 33.6-34.7]; actual mean [SD], 35.8 [1.8] [95% CI, 35.3-36.3]), number of opioid fills per patient (forecasted, 0.49 [95% CI, 0.48-0.51]; actual, 0.54 [95% CI, 0.51-0.55]), and percentage of patients filling a schedule II opioid prescription (forecasted, 28.7% [95% CI, 27.9%-29.6%]; actual, 31.5% [95% CI, 30.6%-32.3%]). There was no significant association with days’ suppy of opioids per prescription or percentage of patients filling a prescription for a schedule III or higher opioid. Results stratified by delivery modality showed that the observed increases were larger for patients who delivered by cesarean birth than those delivering vaginally.

**Conclusions and Relevance:**

This cross-sectional study suggests that the onset of the COVID-19 pandemic was associated with significant increases in postpartum opioid fills. Increases in opioid prescriptions may be associated with increased risk of opioid misuse, opioid use disorder, and opioid-related overdose among postpartum women.

## Key Points

**Question:**

How did fills of opioid prescriptions change among postpartum women after the onset of the COVID-19 pandemic?

**Findings:**

In this cross-sectional study of 460 371 privately insured postpartum women, patients who gave birth to a single, live newborn after March 2020 were more likely to fill more potent and more frequent opioid prescriptions than patients who gave birth prior to March 2020. Increases were larger for patients delivering via cesarean birth than those delivering vaginally.

**Meaning:**

This study suggests that women who gave birth after March 2020 experienced increased exposure to opioids during the postpartum period.

## Teaser

This cross-sectional study of privately insured women compares postpartum opioid prescription fills after the onset of the COVID-19 pandemic in March 2020 with fills before the pandemic.

## Introduction

The postpartum period is a critical period for maternal health care because the incidence of pain and other chronic conditions is high, and more than 50% of maternal deaths occur during the postpartum period.^1^ Disruption to postpartum care by the COVID-19 pandemic has the potential to substantially worsen outcomes for postpartum women and their infants.

One of the most common causes of maternal morbidity during the postpartum period is pain. Approximately two-thirds of women report pain that interferes with activities of daily living at 24 hours post partum, and up to 18% of women experience chronic pain after delivery.^2^ Proper management of postpartum pain is crucial, as evidence shows that untreated pain during the postpartum period increases the risk of postpartum depression and chronic pain.

Current recommendations by the American College of Obstetricians and Gynecologists are for clinicians to follow a stepwise approach to pain management for postpartum women, beginning with acetaminophen and/or nonsteroidal anti-inflammatory drugs and following with low-potency and then high-potency opioids, as needed.^3^ One study from prior to the COVID-19 pandemic found that approximately 45.3% of women received a prescription for opioids at delivery discharge.^4^ Rates of prescribing differed substantially based on delivery modality: 30.4% of women who delivered vaginally and 86.7 % of women who delivered via cesarean birth received an opioid prescription.

Although opioids are very effective for treating pain, the timely receipt of postpartum care is crucial to facilitate appropriate use given their high potential for misuse. American College of Obstetricians and Gynecologists recommendations state that opioid use for treatment of postpartum pain should be for the “shortest reasonable” duration.^5^ However, evidence shows that the number of opioid doses prescribed at delivery are often more than 37% greater than the amount consumed.^6^ In addition, more than 95% of patients do not properly dispose of the excess doses, increasing the potential for misuse. A separate study shows that postpartum opioid prescribing is linked to 21 576 new persistent opioid users annually.^7^ Most postpartum opioid prescribing is to new opioid users,^6^ who may be particularly vulnerable to adverse health effects from opioids.^8^

The broad disruption to health care during the COVID-19 pandemic likely affected the receipt of appropriate care and pain management for postpartum women.^9,10^ During the first several months of the pandemic, overall use of non–COVID-19–related outpatient care decreased by approximately 40%.^9^ In addition, evidence has shown that opioid prescribing patterns changed during the pandemic, with new prescriptions decreasing and the duration of prescriptions increasing.^11,12^ Clinicians treating postpartum women may have responded to expected disruptions to follow-up care by deviating from the recommended stepwise management of pain and increasing the initial strength and duration of opioids prescribed.

To better understand the association of the COVID-19 pandemic with pain-related postpartum health care use, this study examined opioid prescription fills for women in the 6 months after birth. We hypothesized that opioid prescription duration and strength would increase, with the largest changes expected among women who delivered via cesarean birth.

## Methods

### Data Source

Data on all patients with prescriptions of medication for pain were extracted from Optum’s deidentified Clinformatics Data Mart system. Clinformatics is derived from a database of administrative health claims for members of large commercial and Medicare Advantage health plans. These administrative claims are submitted for payment by providers and pharmacies and are verified, adjudicated, adjusted, and deidentified. The database includes approximately 15 million to 20 million annual covered lives, with more than 62 million covered lives over a 12-year period, and is *International Statistical Classification of Diseases and Related Health Problems, Tenth Revision* (*ICD-10*), compliant. We followed the Strengthening the Reporting of Observational Studies in Epidemiology (STROBE) reporting guideline for cross-sectional studies. The University of Georgia institutional review board approved this study and waived the need for informed consent due to the deidentified nature of the data.

This retrospective cross-sectional study includes 460 371 postpartum women and approximately 150 000 live births per year, representing more than 5% of all US births and 14% of births to privately insured women. We observed no Medicaid-insured women in our data. Sample data are aggregated to the patient-birth level and span from July 1, 2018, to December 31, 2020. Inclusion criteria for the sample were (1) patients had at least 6 months of continuous enrollment in their plan, (2) patients in the sample had to have a birth during the observation period, (3) patients with multiple births were excluded (each patient had 1 single live birth), and (4) patients in the sample were at least 18 years of age.

Births and the delivery modality (ie, vaginal or cesarean) were identified from patient medical services files using *Current Procedural Terminology* (*CPT*) and *ICD-10* codes. Both *CPT* and *ICD-10* codes were present in the data for approximately 75% of patients; the remaining patients had only one type of coding structure available (either *CPT* or *ICD-10* code, but not both). Delivery modality was identifiable from the data for most women (99.5% of the sample [460 371 of 462 653]).

In addition to detailing services rendered and procedures, the medical files also include the date of service, which we used to identify the date of delivery. Patient race and ethnicity were available for 81.1% of the sample (373 452 of 460 371). Among commercial administrative data sets, Optum Clinformatics Data Mart reports race and ethnicity using an unvalidated proprietary algorithm derived using member geographic location and name (email communication; Optum Life Science Department Data; August 13, 2021).

For each patient, we merged medical services and prescription files using the unique patient identifier. The prescription file contains information on prescriptions filled by the patient, including the drug name, National Drug Code, prescription strength in milligrams, prescription days’ supply, and fill date. These data do not include drugs administered in the hospital (eg, anesthesia or epidural medication during delivery). To standardize prescription strength across opioids, we used the Centers for Disease Control and Prevention Morphine Milligram Equivalents (MME) datafile, which contains MME conversion factors and US Drug Enforcement Administration (DEA) drug schedule by National Drug Code.^13^

### Outcome Measures

We examined several measures of opioid fills during the postpartum period, defined as the first 6 months after the delivery date. First, for the full sample of women with a single live birth during the observation time frame from July 2018 to December 2020, we used an extensive margin measure of whether the patient filled at least 1 opioid prescription during the postpartum period. Second, we used the following measures to examine the intensity of the opioid prescriptions: total number of filled opioid prescriptions per patient, and, conditional on filling an opioid prescription, the mean number of days supplied and mean strength of the prescriptions per birth. Prescription strength is measured in MMEs per day supplied of the prescription. We also examined whether the patient filled a schedule II opioid prescription or if the patient filled a schedule III or higher prescription. Schedule II drugs are considered by the DEA to have a high potential for abuse; schedule III and higher drugs are considered to have moderate to low potential for abuse and dependence.^14^ Schedule II opioids are also stronger than schedule III or higher opioids in terms of MMEs.^10^ These measures were our outcomes of interest (dependent variables). We also stratified our dependent variables by delivery type, patient age at delivery, and race and ethnicity.

### Statistical Analysis

Statistical analysis was performed from December 1, 2021, to September 15, 2022. Descriptive statistics were calculated for all study variables. Statistical tests were 2-sided. Ordinary least-squares regression was used to fit the time series for each outcome variable using data from births that occurred during the pre–COVID-19 period (July 2018 to February 2020). The regression included indicator variables for each calendar month to capture seasonality in outcomes. A monthly linear trend term was included to control for the preexisting downward trend in postpartum opioid fill rates. The time-series models were then used to forecast expected outcomes for births that occurred during the March 2020 to December 2020 period; 95% CIs were estimated as 1.96 times the SE of the forecast, above or below our forecast. Forecasted and actual values were then compared to assess the association of the COVID-19 pandemic with opioid prescription fills among postpartum women.

All descriptive statistics and ordinary least-squares estimations were estimated for the full sample of women as well as stratified by delivery type (vaginal or cesarean delivery). Data were analyzed using Stata, version 17.0 (StataCorp LLC).

## Results

Opioid fills were analyzed for 460 371 patients (mean [SD] age at delivery, 29.0 [10.8] years). Over the study period, a mean (SD) of 38.1% (48.6%) of all women in the sample filled an opioid prescription in the postpartum period, with 23.8% (42.6%) of women with vaginal deliveries filling at least 1 opioid prescription and 67.0% (47.0%) of women with cesarean deliveries filling at least 1 opioid prescription (Table). The mean (SD) number of opioid prescriptions per person was 0.5 (1.0) for all women, 0.3 (0.8) for women with vaginal deliveries, and 0.9 (1.1) for women with cesarean deliveries. Among women who filled an opioid prescription, women received a mean (SD) of 35.0 (12.3) MMEs of opioids per day (32.7 [11.9] MMEs for women with vaginal delivery and 36.6 [12.3] MMES for those with cesarean delivery).

**Table.** Descriptive Statistics of Patients and Opioid Prescriptions, July 2018 to December 2020

Characteristic	Mean (SD) value		
	Full sample (N = 460 371)	Vaginal delivery (n = 307 671)	Cesarean delivery (n = 152 700)
Age at delivery, y	29.0 (10.8)	28.6 (10.0)	29.6 (12.2)
Ever receive an opioid prescription, %	38.1 (48.6)	23.8 (42.6)	67.0 (47.0)
No. of opioid prescriptions per person	0.5 (1.0)	0.3 (0.8)	0.9 (1.1)
MMEs per day, conditional on receiving an opioid prescription	35.0 (12.3)	32.7 (11.9)	36.6 (12.3)
Days’ supply, conditional on receiving an opioid prescription	4.4 (2.4)	4.1 (2.6)	4.7 (2.2)
Patients receiving a schedule II opioid, %	35.1 (47.7)	21.1 (40.8)	63.2 (48.2)
Patients receiving a schedule III or higher opioid, %	3.7 (18.9)	3.2 (17.6)	4.7 (21.2)

Abbreviation: MMEs, morphine milligram equivalents.

Conditional on filling an opioid prescription, the mean (SD) days’ supply of opioids was 4.4 (2.4) days over the study period. The mean (SD) days’ supply was higher among women with cesarean deliveries (4.7 [2.2]) than those with vaginal deliveries (4.1 [2.6]). Overall, fills of schedule II opioids were more frequent than fills of schedule III or higher opioids, with 35.1% of all women filling a schedule II opioid and 3.7% of women filling a schedule III or higher opioid. Finally, fills of schedule II and schedule III or higher opioids were greater among women with cesarean deliveries (schedule II, 63.2% and schedule III, 4.7%), compared with women with vaginal deliveries (schedule II, 21.1% and schedule III, 3.2%). Descriptive statistics for the subsample of patients with race and ethnicity information are in eTable 1 in Supplement 1.

**Figure 1.**
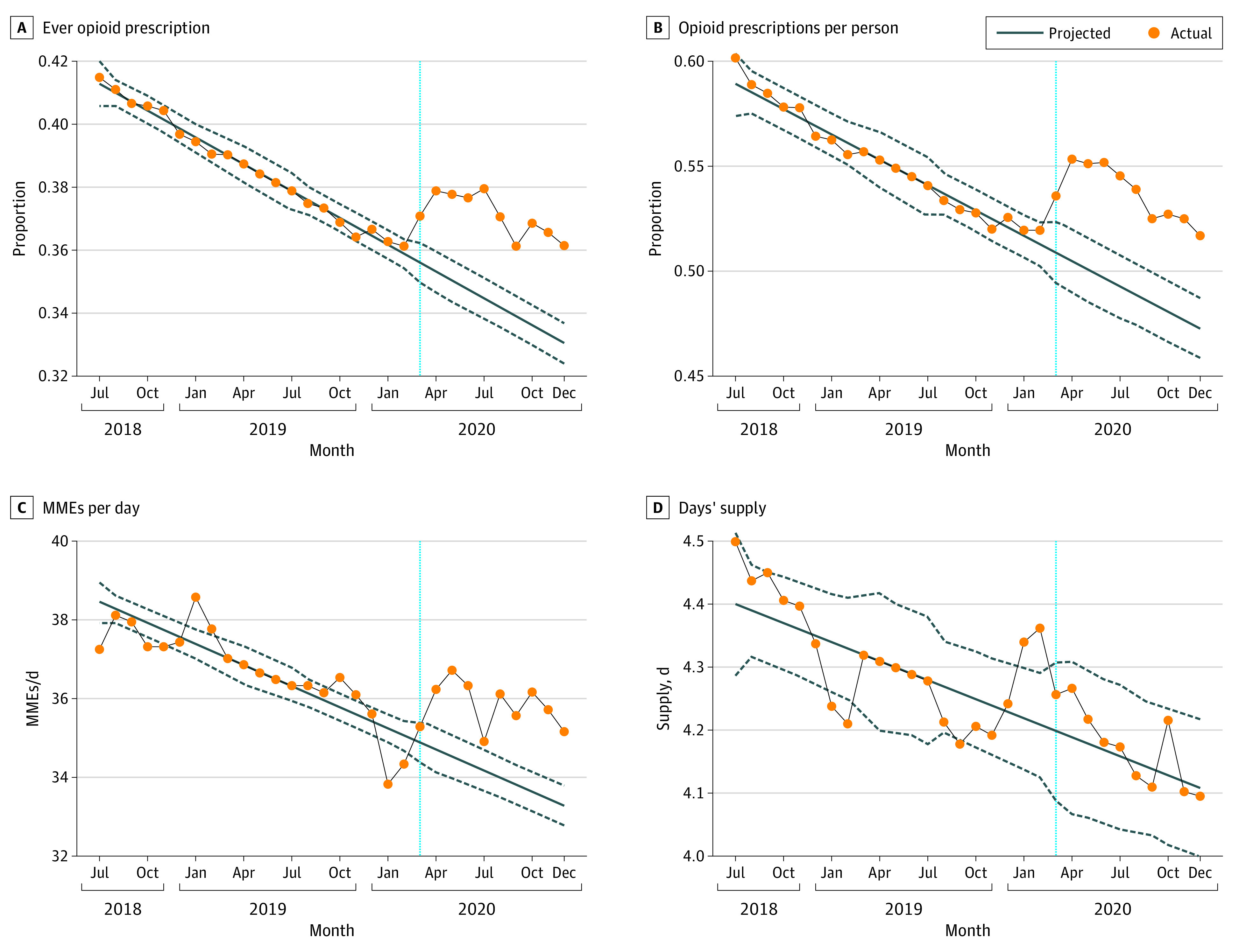
Comparison of Percentage of Patients Receiving an Opioid and Variables of Intensity: All Delivery Types, Pre– and Post–COVID-19 Onset in March 2020 A, Share of postpartum women filling an opioid prescription in a given month. B, Mean number of opioid prescriptions per woman. C, Mean morphine milligram equivalents (MMEs) per day supplied, conditional on filling an opioid prescription. D, Mean days’ supply, conditional on filling an opioid prescription. Each dot represents the measure over a monthly period. The solid diagonal lines and dashed lines represent the forecasted values from our time-series model and the associated 95% CIs, respectively. The vertical dotted blue line corresponds to March 2020.

Figure 1 shows the deseasonalized actual and forecasted values for the proportion of postpartum women filling at least 1 opioid prescription before and after the initial onset of the COVID-19 pandemic (March 2020), as well as separately for 3 measures of prescription intensity: number of opioid fills per patient, mean MMEs per day, and mean days’ supply. Postpartum women were significantly more likely to fill an opioid prescription during the COVID-19 period than expected based on the preexisting time trend. On average across all months after March 2020, the actual rate of postpartum women filling any opioid prescription was 2.8 percentage points higher than forecasted (forecasted, 35.0% [95% CI, 34.0%-35.9%]; actual, 37.8% [95% CI, 36.8%-38.7%]). There was also an increase of 4.6 opioid fills per 100 women, or 0.05 fills per patient, in the COVID-19 period (forecasted, 0.49 [95% CI, 0.48-0.51]; actual, 0.54 [95% CI, 0.51-0.55]). In addition, among women who filled an opioid prescription, prescription strength increased by 1.7 MMEs per day in the COVID-19 period (forecasted, 34.1 [95% CI, 33.6-34.6]; actual, 35.8 [95% CI, 35.3%-36.3%]). However, conditional on filling an opioid prescription, there was no difference between the actual and forecasted values for the mean days’ supply. See eTable 2 in Supplement 1 for deseasonalized actual and forecasted values with 95% CIs by month.

**Figure 2.**
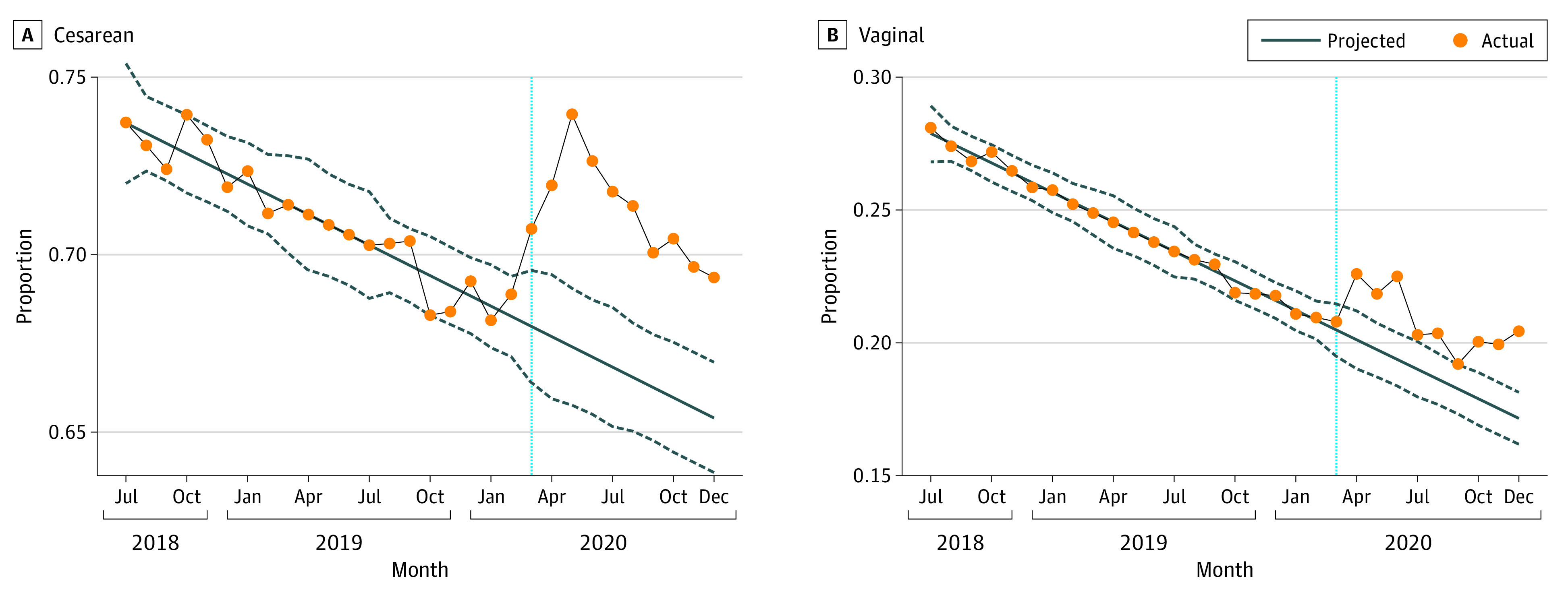
Comparison of Percentage of Patients Receiving an Opioid, by Delivery Type, Pre– and Post–COVID-19 Onset in March 2020 A, Share of postpartum women with a cesarean delivery filling an opioid prescription in a given month. B, Share of postpartum women with a vaginal delivery filling an opioid prescription in a given month. Each dot represents the measure over a monthly period. The solid diagonal lines and dashed lines represent the forecasted values from our time-series model and the associated 95% CIs, respectively. The vertical dotted blue line corresponds to March 2020.

In Figure 2, results indicate that the difference between actual and forecasted opioid fill rates was higher among women with cesarean deliveries (4.5 percentage points; forecasted, 66.7% [95% CI, 65.1%-68.3%]; actual, 71.2% [95% CI, 69.6%-72.8%]) compared with women with vaginal deliveries (2.0 percentage points; forecasted, 18.8% [95% CI, 17.8%-19.8%]; actual, 20.8% [95% CI, 19.8%-21.8%]). Monthly deseasonalized actual and forecasted values with 95% CIs are presented in eTable 3 in Supplement 1. Relative to the mean rates of opioid fills for each delivery modality, this represents a 6.7% increase for cesarean deliveries, compared with an 8.4% increase for vaginal deliveries. eFigure 1 in Supplement 1 shows that there is no evidence of a coincident increase in the probability of having a cesarean birth during the COVID-19 period.

Results by delivery type for measures of opioid prescription intensity indicate larger increases in MMEs per day and in the number of opioid fills per person among women with cesarean deliveries during the COVID-19 period (eTable 4 in Supplement 1). Among women who filled an opioid prescription, there was an increase of 1.9 MMEs per day (forecasted, 35.6 [95% CI, 34.9-36.3]; actual, 37.5 [95% CI, 36.8%-38.2%]) compared with 1.3 MMEs per day among women with vaginal deliveries in the COVID-19 period (forecasted, 31.4 [95% CI, 30.6-32.2]; actual, 32.7 [95% CI, 31.9%-33.5%]). Women with cesarean deliveries showed an increase of 8.8 opioid fills per 100 patients (difference, 0.09 per patient; forecasted, 0.90 [95% CI, 0.86-0.93]; actual, 0.98 [95% CI, 0.95-1.02]) compared with an increase of 3.3 opioid fills per 100 patients for women with vaginal deliveries (difference, 0.03 per patient; forecasted, 0.26 [95% CI, 0.24-0.28]; actual, 0.29 [95% CI, 0.27-0.31]) during the COVID-19 period.

Results by maternal age at delivery and race and ethnicity are presented in eFigures 2 and 3 in Supplement 1, respectively. The onset of the COVID-19 pandemic was associated with larger increases in opioid fills for women aged 18 to 30 years (3.1 percentage points; forecasted, 30.9% [95% CI, 29.8%-31.9%]; actual, 34.0% [95% CI, 32.9%-35.0%]) and 31 to 40 years (2.7 percentage points; forecasted, 36.1% [95% CI, 35.4%-37.0%]; actual, 38.8% [95% CI, 37.9%-39.7%]) at the time of delivery relative to women who were 41 years or older (1.0 percentage points; forecasted, 41.2% [95% CI, 37.7%-44.6%]; actual, 42.2% [95% CI, 38.7%-45.6%]). Analyses by maternal race and ethnicity show that Black women experienced smaller increases in opioid fills (2.1 percentage points; forecasted, 41.0% [95% CI, 38.8%-43.2%]; actual, 43.1% [95% CI, 40.9%-45.4%]) relative to White women (3.1 percentage points; forecasted, 34.1% [95% CI, 33.2%-34.9%]; actual, 37.2% [95% CI, 36.4%-38.1%]) or Asian and Latinx or Hispanic women (forecasted, 32.2% [95% CI, 30.7%-33.6%]; actual, 34.8% [95% CI, 33.3%-36.2%]).

**Figure 3.**
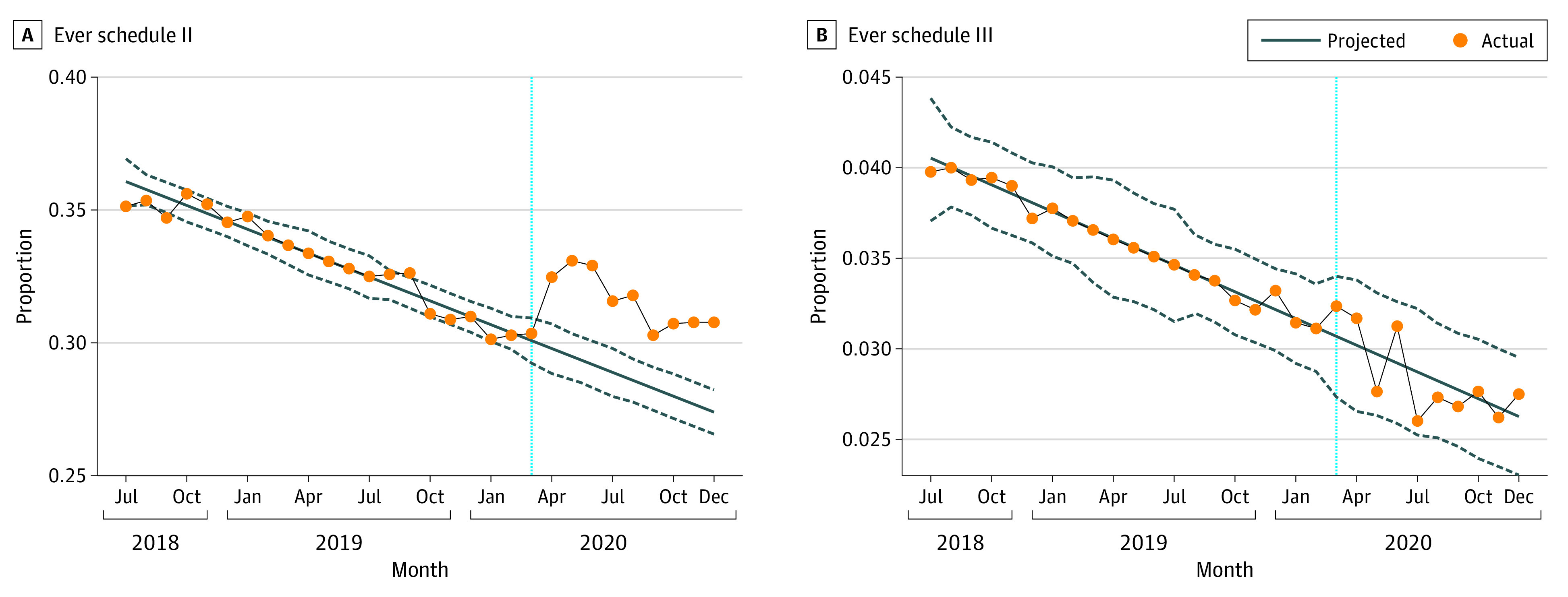
Comparison of Prescribing by Drug Enforcement Administration Drug Schedule: All Delivery Types, Pre– and Post–COVID-19 Onset in March 2020 A, Share of postpartum women filling a prescription for a schedule II opioid in a given month. B, Share of postpartum women filling a prescription for a schedule III or higher opioid in a given month. Each dot represents the measure over a monthly period. The solid diagonal lines and dashed lines represent the forecasted values from our time-series model and the associated 95% CIs, respectively. The vertical dotted blue line corresponds to March 2020.

Figure 3 shows that the overall increase in opioid fills occurred due to an increase in fills of schedule II opioids. The proportion of patients ever filling a schedule II opioid increased by 2.8 percentage points (forecasted, 28.7% [95% CI, 27.9%-29.6%]; actual, 31.5% [95% CI, 30.6%-32.3%]). There was no statistically significant change in the number of patients filling a schedule III or higher opioid. See eTable 5 in Supplement 1 for monthly deseasonalized actual and forecasted values with 95% CIs.

## Discussion

Our results indicate that after a year and a half of consistent decreases,^5^ postpartum opioid fills significantly increased at the start of the COVID-19 pandemic, and this increase was sustained through at least December 2020. Across all delivery types, we found a 2.8-percentage point higher opioid fill rate, 1.7 higher MMEs per day per prescription, 4.6 more opioid prescriptions filled per 100 patients, and a 2.8-percentage point higher prescription rate of schedule II opioids during the COVID-19 period than forecasted based on preexisting trends. We did not find a significant change in the days’ supply of opioid prescriptions. The observed increases were larger for women who give birth via cesarean delivery than those delivering vaginally.

These results showed that after the onset of the COVID-19 pandemic, more postpartum women were exposed to opioids, they filled more opioid prescriptions, and the strength of those prescriptions was higher. Furthermore, while prescription fills of schedule III or higher opioids continued to decline during this period, fills of schedule II opioids, which are stronger and considered to carry a higher risk of abuse, increased. Previous studies examining the association of COVID-19 with opioid prescribing have examined broader patient populations, without focusing on postpartum women.^11,12^ Postpartum women are a particularly important population to study, given high rates of severe pain,^2^ and the fact that most postpartum opioid prescribing is to new opioid users,^6^ who may be particularly vulnerable to adverse health effects from opioids.^8^ Our results run counter to existing evidence that opioid prescribing to opioid-naive patients declined during the early months of the COVID-19 pandemic.^12^ However, our results are consistent with evidence that, conditional on receiving a diagnosis of pain, during the COVID-19 pandemic patients were more likely to receive an opioid prescription and those opioid prescriptions were relatively more potent.^11^

These findings have numerous implications for health care professionals and policy makers. Poor management of postpartum pain can increase the risk of postpartum depression and chronic pain; thus, increased opioid use may represent more appropriate treatment of severe pain for some postpartum women. This may be particularly true for racial and ethnic minority individuals, who are often undertreated for pain relative to White individuals in the US.^15,16,17,18,19,20,21^

On the other hand, overprescription of opioids can lead to persistent misuse.^6,22^ This potential for misuse was compounded during COVID-19, as the isolation and stressors of the pandemic may have been associated with women misusing opioids as a coping mechanism.^23^ The disruption COVID-19 caused to health care for postpartum women could mean that while clinicians were prescribing more potent opioids more frequently in hopes of preventing excessive postpartum pain, if their patients develop an opioid use disorder (OUD) it will take longer to diagnose and treat.^9^ Evidence shows that initiation of buprenorphine treatment for OUD declined during the early pandemic months,^12^ and postpartum women with OUD experience additional barriers to treatment, including stigma and fear of legal consequences and child welfare involvement.^24^ Also, individuals with OUD experience increased risk of COVID-19,^25^ which can present additional challenges for postpartum women as they care for themselves and their newborns.

Our findings are particularly concerning given that the COVID-19 pandemic has exacerbated the effects of the US opioid crisis. Monthly opioid-related overdose deaths sharply increased after the March 23, 2020, stay-at-home order. In addition, opioid-related overdose deaths reached an all-time high during the pandemic, surpassing 100 000 annually.^26,27^

### Limitations

This study has several limitations. First, in the prescription database files, we did not differentiate between initial fills and refills. Second, we did not observe prescriptions that the clinician prescribed but the patient did not fill. Thus, we do not know whether the change in opioid prescriptions filled during the COVID-19 period was due to changes in prescriber behavior or patient decisions to fill opioid prescriptions. Third, for a small percentage of women in the sample (<10%), we did not observe the mode of delivery because the clinician used a global *CPT* code that indicated a single live birth but did not distinguish between vaginal or cesarean delivery. These deliveries were omitted from analysis. Fourth, because patients younger than 18 years were excluded from the sample, these results are not generalizable to women younger than 18 years who gave birth during this time period.

## Conclusions

This retrospective cross-sectional study found that the onset of the COVID-19 pandemic was associated with significant increases in postpartum opioid fills. In particular, we found an increase in the frequency and potency of filled opioid prescriptions. Given concerns of potential misuse and other harms associated with opioid use for postpartum women, it is important to understand the short- and long-term associations of these changes with fill patterns. Future research should examine whether changes in postpartum opioid fills after the onset of the COVID-19 pandemic have continued and whether these changes have translated into increases in opioid misuse, OUD, and opioid-related overdose among postpartum women.

## Notes

Supplement 1. **eTable 1.** Means for All Delivery Types: by Patient Race/Ethnicity **eFigure 1.** Rate of Cesarean Births Pre- and Post–COVID-19 Onset in March 2020 **eTable 2.** Deseasonalized Forecasted and Actual Values by Month: All Delivery Types **eTable 3.** Ever Opioid Rx-Deseasonalized Forecasted and Actual Values by Month: by Delivery Type **eTable 4.** Schedule II and Schedule III or Higher Opioid Rx-Deseasonalized Forecasted and Actual Values by Month: All Delivery Types **eFigure 2.** Rate of Ever Opioid Fills Pre- and Post–COVID-19 Onset in March 2020: by Maternal Age **eFigure 3.** Rate of Ever Opioid Fills Pre- and Post–COVID-19 Onset in March 2020: by Maternal Race/Ethnicity **eTable 5.** Deseasonalized Forecasted and Actual Values by Month: by Delivery Type

Supplement 2.
Data Sharing Statement
