# Supplementary material for: Comparison of Postpartum Opioid Prescriptions Before vs During the COVID-19 Pandemic
Source: JAMA Netw Open. 2023 Apr 3;6(4):e236438. doi: 10.1001/jamanetworkopen.2023.6438 (PMC10071338; doi:10.1001/jamanetworkopen.2023.6438)
Supplement: Supplement 1. — eTable 1. Means for All Delivery Types: by Patient Race/Ethnicity eFigure 1. Rate of Cesarean Births Pre- and Post–COVID-19 Onset in March 2020 eTable 2. Deseasonalized Forecasted and Actual Values by Month: All Delivery Types eTable 3. Ever Opioid Rx-Deseasonalized Forecasted and Actual Values by Month: by Delivery Type eTable 4. Schedule II and Schedule III or Higher Opioid Rx-Deseasonalized Forecasted and Actual Values by Month: All Delivery Types eFigure 2. Rate of Ever Opioid Fills Pre- and Post–COVID-19 Onset in March 2020: by Maternal Age eFigure 3. Rate of Ever Opioid Fills Pre- and Post–COVID-19 Onset in March 2020: by Maternal Race/Ethnicity eTable 5. Deseasonalized Forecasted and Actual Values by Month: by Delivery Type [file jamanetwopen-e236438-s001.pdf]

Supplementary Online Content

Steuart SR, Lawler EC, Bagwell Adams G, Shone H, Abraham AJ. Comparison of postpartum opioid prescriptions before vs during the COVID-19 pandemic. *JAMA Netw Open.* 2023;6(4):e236438. doi:10.1001/jamanetworkopen.2023.6438

eTable 1. Means for All Delivery Types: by Patient Race/Ethnicity
eFigure 1. Rate of Cesarean Births Pre- and Post–COVID-19 Onset in March 2020
eTable 2. Deseasonalized Forecasted and Actual Values by Month: All Delivery Types
eTable 3. Ever Opioid Rx-Deseasonalized Forecasted and Actual Values by Month: by Delivery Type
eTable 4. Schedule II and Schedule III or Higher Opioid Rx-Deseasonalized Forecasted and Actual Values by Month: All Delivery Types
eFigure 2. Rate of Ever Opioid Fills Pre- and Post–COVID-19 Onset in March 2020: by Maternal Age
eFigure 3. Rate of Ever Opioid Fills Pre- and Post–COVID-19 Onset in March 2020: by Maternal Race/Ethnicity
eTable 5. Deseasonalized Forecasted and Actual Values by Month: by Delivery Type

This supplementary material has been provided by the authors to give readers additional information about their work.

© 2023 Steuart SR et al. *JAMA Network Open.*

eTable 1. Means for All Delivery Types: by Patient Race/Ethnicity

	(1) Full Sample	(2) All Patients with Race/Ethnicity Variable	(3) White Patients	(4) Black Patients	(5) Asian and Hispanic/ Latinx Patients
Average Age at Delivery	28.953	31.887	31.887	31.350	32.130
Share White Patients	0.550	0.678	1	0	0
Share Black Patients	0.082	0.101	0	1	0
Share Missing Race/Ethnicity Variable	0.189	0	0	0	0
Ever Received Opioid Rx	0.381	0.420	0.421	0.464	0.396
Avg # of Opioid Rxs per Person	0.513	0.568	0.578	0.641	0.506
Avg MMEs per day, Conditional on Receiving an opioid Rx	34.990	35.066	35.429	35.108	33.934
Avg Days Supplied, Conditional on Receiving an Opioid Rx	4.437	4.449	4.387	4.514	4.603
Percent of Patients Receiving a Schedule II Opioid	0.351	0.386	0.393	0.426	0.347
Percent of Patients Receiving a Schedule III+ Opioid	0.037	0.041	0.036	0.049	0.055
Percent of Patients with Vaginal Delivery	0.668	0.672	0.686	0.639	0.647
Percent of Patients with Cesarean Delivery	0.332	0.328	0.314	0.361	0.353
Observations	460371	373452	253103	37582	82767

© 2023 Steuart SR et al. *JAMA Network Open.*

eFigure 1. Rate of Cesarean Births Pre- and Post–COVID-19 Onset in March 2020

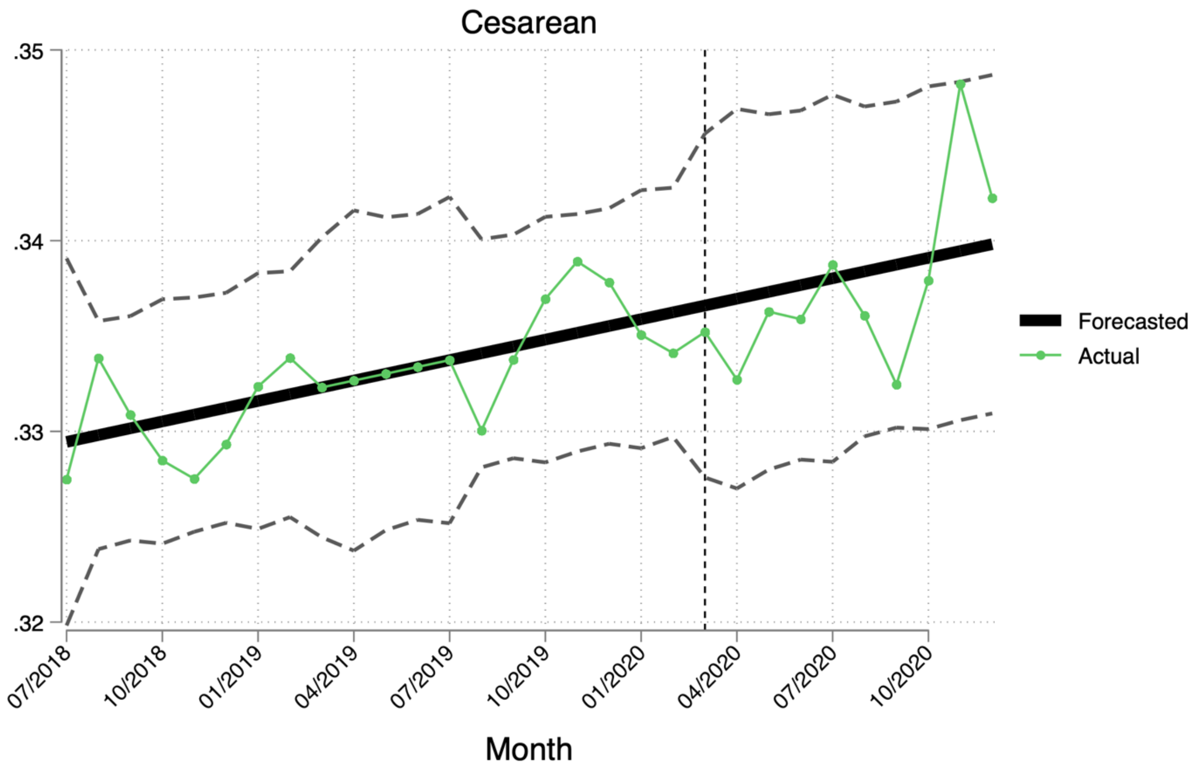

Share of births that were delivered via Cesarean section. Each green dot represents the measure over a monthly period. The solid black points and dashed gray lines represent the forecasted values from our time-series model and the associated 95 percent confidence interval, respectively. Labels on the x-axes refer to the month; the vertical solid black line corresponds to March 2020.

© 2023 Steuart SR et al. *JAMA Network Open.*

eTable 2. Deseasonalized Forecasted and Actual Values by Month: All Delivery Types

	Month of 2020	Deseasonalized Actual Value	Forecasted Value	Confidence Interval [LB,	UB]	Difference
Ever Opioid Rx	March	0.375	0.364	[0.355,	0.374]	0.011
	April	0.390	0.361	[0.351,	0.371]	0.029
	May	0.393	0.358	[0.348,	0.368]	0.035
	June	0.393	0.355	[0.345,	0.364]	0.038
	July	0.377	0.351	[0.342,	0.361]	0.026
	August	0.375	0.348	[0.339,	0.357]	0.027
	September	0.361	0.345	[0.336,	0.354]	0.016
	October	0.371	0.342	[0.332,	0.351]	0.029
	November	0.372	0.338	[0.329,	0.348]	0.034
	December	0.371	0.335	[0.326,	0.344]	0.036
Opioid Rx per Person	March	0.536	0.509	[0.494,	0.523]	0.027
	April	0.554	0.505	[0.490,	0.520]	0.049
	May	0.551	0.501	[0.486,	0.516]	0.050
	June	0.552	0.497	[0.482,	0.512]	0.056
	July	0.546	0.493	[0.477,	0.508]	0.053
	August	0.540	0.489	[0.475,	0.503]	0.051
	September	0.525	0.485	[0.471,	0.499]	0.040
	October	0.527	0.481	[0.466,	0.495]	0.047
	November	0.525	0.477	[0.462,	0.491]	0.049
	December	0.518	0.473	[0.458,	0.487]	0.045

© 2023 Steuart SR et al. *JAMA Network Open.*

eTable 2. Deseasonalized Forecasted and Actual Values by Month: All Delivery Types (continued)

	Month of 2020	Deseasonalized Actual Value	Forecasted Value	Confidence Interval [LB, UB]		Difference
MMEs per Day	March	35.304	34.937	[34.429,	35.445]	0.367
	April	36.275	34.760	[34.185,	35.334]	1.515
	May	36.651	34.582	[34.045,	35.120]	2.069
	June	36.251	34.405	[33.877,	34.933]	1.846
	July	34.792	34.228	[33.681,	34.774]	0.565
	August	36.195	34.050	[33.552,	34.549]	2.144
	September	35.553	33.873	[33.379,	34.367]	1.681
	October	36.071	33.695	[33.178,	34.213]	2.375
	November	35.910	33.518	[33.004,	34.032]	2.392
	December	35.115	33.341	[32.823,	33.859]	1.775
Day Supply	March	4.164	4.113	[4.012,	4.213]	0.052
	April	4.128	4.101	[3.987,	4.214]	0.028
	May	4.086	4.089	[3.982,	4.195]	-0.002
	June	4.072	4.077	[3.972,	4.181]	-0.004
	July	4.037	4.064	[3.956,	4.172]	-0.028
	August	4.063	4.052	[3.954,	4.151]	0.011
	September	3.980	4.040	[3.943,	4.138]	-0.060
	October	4.073	4.028	[3.926,	4.131]	0.045
	November	4.011	4.016	[3.915,	4.118]	-0.005
	December	4.031	4.004	[3.902,	4.107]	0.026

© 2023 Steuart SR et al. *JAMA Network Open.*

eTable 3. Ever Opioid Rx-Deseasonalized Forecasted and Actual Values by Month: by Delivery Type

	Month of 2020	Deseasonalized Actual Value	Forecasted Value	Confidence Interval [LB,	UB]	Difference
Vaginal Deliveries	March	0.208	0.205	[0.195,	0.215]	0.003
	April	0.226	0.201	[0.190,	0.212]	0.025
	May	0.219	0.197	[0.187,	0.208]	0.021
	June	0.225	0.194	[0.184,	0.204]	0.031
	July	0.203	0.190	[0.179,	0.201]	0.013
	August	0.204	0.186	[0.177,	0.196]	0.018
	September	0.192	0.183	[0.173,	0.192]	0.009
	October	0.200	0.179	[0.169,	0.189]	0.021
	November	0.199	0.175	[0.165,	0.185]	0.024
	December	0.204	0.172	[0.162,	0.181]	0.033
Cesarean Deliveries	March	0.707	0.680	[0.664,	0.696]	0.027
	April	0.720	0.677	[0.659,	0.694]	0.043
	May	0.740	0.674	[0.658,	0.690]	0.066
	June	0.726	0.671	[0.655,	0.687]	0.055
	July	0.718	0.668	[0.651,	0.685]	0.049
	August	0.714	0.665	[0.650,	0.681]	0.048
	September	0.701	0.663	[0.648,	0.678]	0.038
	October	0.704	0.660	[0.644,	0.675]	0.045
	November	0.697	0.657	[0.641,	0.672]	0.040
	December	0.694	0.654	[0.639,	0.669]	0.040

© 2023 Steuart SR et al. *JAMA Network Open.*

eTable 4. Schedule II and Schedule III or Higher Opioid Rx-Deseasonalized Forecasted and Actual Values by Month: All Delivery Types

	Month of 2020	Deseasonalized Actual Value	Forecasted Value	Confidence Interval		Difference
				[LB,	UB]	
Schedule II	March	0.303	0.301	[0.292,	0.309]	0.003
	April	0.324	0.298	[0.288,	0.307]	0.027
	May	0.331	0.295	[0.286,	0.303]	0.036
	June	0.329	0.292	[0.283,	0.300]	0.037
	July	0.316	0.289	[0.280,	0.298]	0.027
	August	0.318	0.286	[0.278,	0.294]	0.032
	September	0.303	0.283	[0.275,	0.291]	0.020
	October	0.307	0.280	[0.271,	0.288]	0.027
	November	0.308	0.277	[0.269,	0.285]	0.031
	December	0.308	0.274	[0.265,	0.282]	0.034
Schedule III+	March	0.090	0.083	[0.076,	0.091]	0.006
	April	0.090	0.083	[0.075,	0.090]	0.007
	May	0.082	0.082	[0.074,	0.089]	0.000
	June	0.084	0.081	[0.073,	0.089]	0.003
	July	0.076	0.080	[0.072,	0.088]	-0.004
	August	0.078	0.079	[0.072,	0.086]	-0.002
	September	0.075	0.078	[0.071,	0.086]	-0.003
	October	0.079	0.077	[0.070,	0.085]	0.001
	November	0.075	0.077	[0.069,	0.084]	-0.001
	December	0.085	0.076	[0.068,	0.083]	0.009

© 2023 Steuart SR et al. *JAMA Network Open.*

eFigure 2. Rate of Ever Opioid Fills Pre- and Post–COVID-19 Onset in March 2020: by Maternal Age

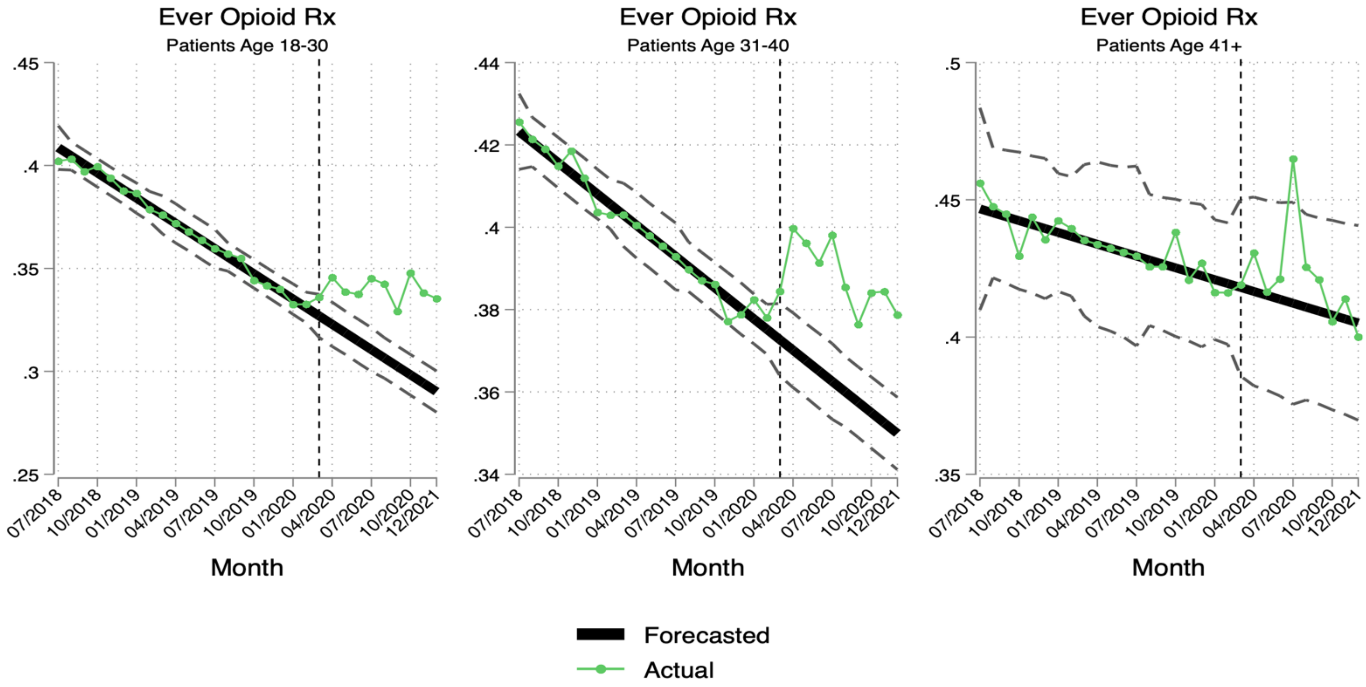

Share of postpartum women age 18-30 filling an opioid prescription in a given month (top panel), share of postpartum women age 31-40 filling an opioid prescription (middle panel), and share of postpartum women age 41+ filling an opioid prescription (middle panel). Each green dot represents the measure over a monthly period. The solid black points and dashed gray lines represent the forecasted values from our time-series model and the associated 95 percent confidence interval, respectively. Labels on the x-axes refer to the month; the vertical solid black line corresponds to March 2020.

© 2023 Steuart SR et al. *JAMA Network Open.*

eFigure 3. Rate of Ever Opioid Fills Pre- and Post–COVID-19 Onset in March 2020: by Maternal Race/Ethnicity

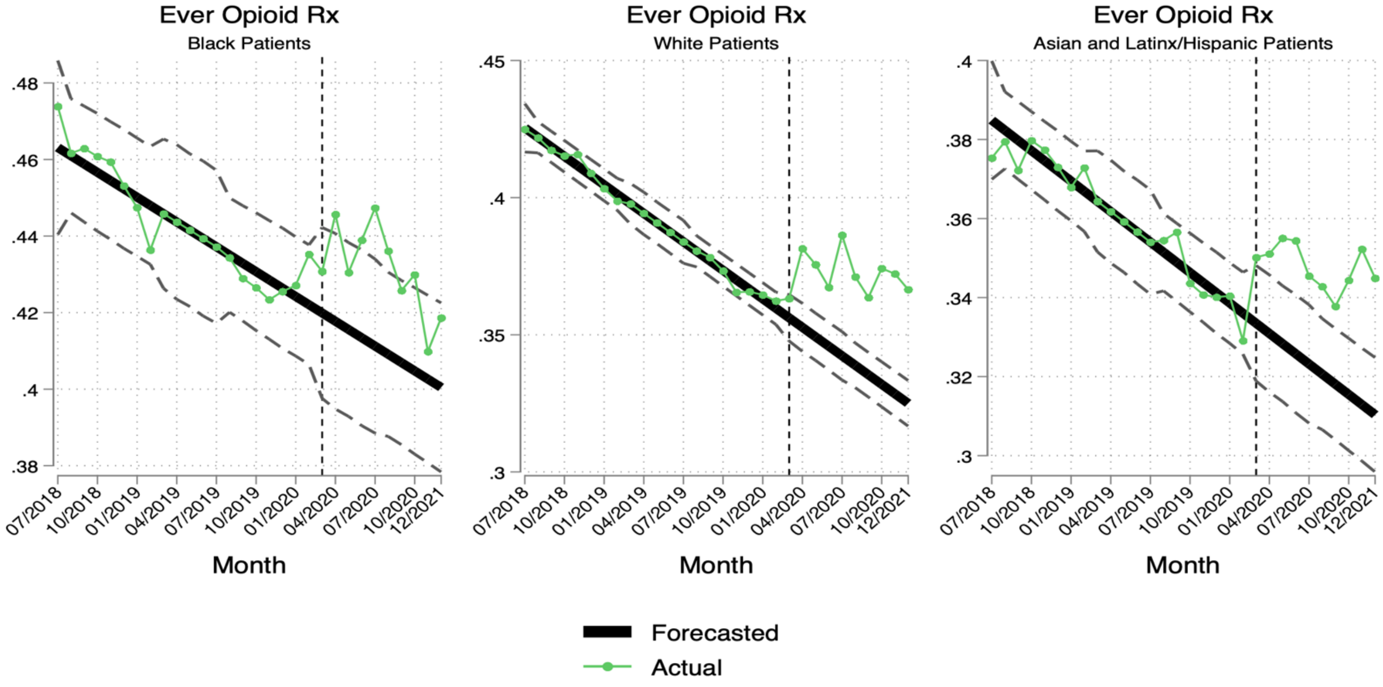

Share of Black postpartum women filling an opioid prescription in a given month (top panel), share of White postpartum women filling an opioid prescription (middle panel), and share of Asian and Latinx/Hispanic postpartum women filling an opioid prescription (middle panel). Each green dot represents the measure over a monthly period. The solid black points and dashed gray lines represent the forecasted values from our time-series model and the associated 95 percent confidence interval, respectively. Labels on the x-axes refer to the month; the vertical solid black line corresponds to March 2020.

© 2023 Steuart SR et al. *JAMA Network Open.*

eTable 5. Deseasonalized Forecasted and Actual Values by Month: by Delivery Type

		Deseasonalized Actual Value	Forecasted Value	Confidence Interval		Difference
				LB	UB	
Cesarean	Ever Opioid Rx	0.712	0.667	0.651	0.683	0.045
	MMEs per Day	37.495	35.576	34.897	36.255	1.919
	Opioid Rxs per Person	0.984	0.896	0.858	0.934	0.088
	Days Supply	4.226	4.237	4.115	4.359	-0.011
	Schedule II	0.568	0.517	0.501	0.532	0.051
	Schedule III+	0.040	0.042	0.036	0.048	-0.002
Vaginal	Ever Opioid Rx	0.208	0.188	0.178	0.198	0.020
	MMEs per Day	32.733	31.397	30.601	32.192	1.337
	Opioid Rxs per Person	0.289	0.256	0.236	0.275	0.033
	Days Supply	3.744	3.700	3.521	3.878	0.044
	Schedule II	0.174	0.156	0.147	0.166	0.017
	Schedule III+	0.022	0.022	0.018	0.026	0.001

© 2023 Steuart SR et al. *JAMA Network Open.*
