# Supplementary material for: Comparison of Postpartum Opioid Prescriptions Before vs During the COVID-19 Pandemic
Source: JAMA Netw Open. 2023 Apr 3;6(4):e236438. doi: 10.1001/jamanetworkopen.2023.6438 (PMC10071338; doi:10.1001/jamanetworkopen.2023.6438)
Supplement: Supplement 2. — Data Sharing Statement [file jamanetwopen-e236438-s002.pdf]

Data Sharing Statement

Steuart. Comparison of Postpartum Opioid Prescriptions Before vs During the COVID-19 Pandemic. *JAMA Netw Open*. Published April 03, 2023.
doi:10.1001/jamanetworkopen.2023.6438

Data
Data available: No

Additional Information
Explanation for why data not available: We use proprietary data that we must keep private as per our data use agreement.
